# Relationships of mobile phone duration and unlock counts with sleep: double machine learning and traditional statistical methods

**DOI:** 10.3389/fpubh.2026.1898932

**Published:** 2026-07-15

**Authors:** Lei Zhang, Mingyang Wu, Zhe Wang, Xue Wang, Xiaoxiao Yuan, Le Ma, Wenhua Wang

**Affiliations:** 1 School of General Medicine, Xi’an Medical University, Xi’an, China; 2 Shaanxi Provincial Health Industry Association Service Center, Xi’an, China; 3 Shaanxi Medical Association, Xi’an, China; 4 Xiangya School of Public Health, Central South University, Changsha, China; 5 School of Public Health, Xi’an Jiaotong University Health Science Center, Xi’an, China

**Keywords:** double machine learning, mobile phone duration, mobile phone unlock counts, sleep quality, sleep time

## Abstract

**Background:**

The association between mobile phone use and sleep is still contested, especially for youth. While most existing studies predominantly rely on self-reported smartphone duration using traditional statistical methods, the potential association between mobile phone unlock counts and sleep has been neglected in prior studies primarily due to the difficulty of estimating the data. This relationship has been largely overlooked due to objective measurement challenges.

**Aims:**

To examine the associations of mobile phone duration and unlock counts with sleep quality and sleep time.

**Methods:**

This multi-center investigation included 16,668 participants from six Chinese universities. Objective mobile phone duration and unlock counts were assessed by mobile phone use record screenshots. The Pittsburgh Sleep Quality Index was used to assess sleep quality and time. Double Machine Learning (DML), linear regression, and restricted cubic splines (RCS) models were applied.

**Results:**

DML revealed that a 7-h/week increase in mobile phone duration was associated with higher PSQI scores (*ATE* = 0.03, *95% CI*: 0.02 to 0.03) and shorter sleep time (*ATE* = −4.76, *95%CI*: −6.13 to −3.65). Similarly, a 50-time/week increase in unlock counts was linked to increased PSQI scores (*ATE* = 0.42, *95%CI*: 0.21 to 0.63) and reduced sleep time (*ATE* = −0.89, *95%CI*: −1.32 to −0.46). RCS models identified non-linear associations of mobile phone duration with poor sleep and sleep time, notably an inverted U-shaped relationship with sleep time (*P* for non-linearity < 0.001). Mobile phone unlock counts showed a significant non-linear association with odds of poor sleep (*P* for non-linearity < 0.01), but a predominantly monotonic decline with sleep time (*P* for non-linearity = 0.50).

**Conclusion:**

Both mobile phone duration and unlock counts are associated with sleep quality and sleep time. Mobile phone duration exhibited non-linear associations with sleep time and poor sleep, showing an inverted U-shaped relationship with sleep time. Mobile phone unlock counts exhibited a non-linear relationship with sleep time, but a predominantly monotonic inverse relationship with odds of poor sleep.

## 1 Introduction

Sufficient and high-quality sleep is fundamental to sustaining physiological and psychological well-being. Recent research revealed a concerning escalation of the rising trend toward poor sleep quality and inadequate duration, with nearly one-third of people worldwide not reaching the internationally endorsed nocturnal sleep requirement of 7 ~ 9 h (1). These cumulative sleep problems demonstrate robust associations with elevated susceptibility to adverse health outcomes (2–4). In the digital age, the effect of mobile phone use on sleep is still contested, particularly among youth. As digital natives, this generation of youth devotes substantially more time to mobile phone interactions than previous generations, making mobile phones an indispensable part of daily life. Earlier studies suggest that mobile phones can influence sleep through several pathways, including sleep displacement from mobile phone screen exposure (5), the alerting effects of screen-emitted light on the circadian system (6), and interference with falling asleep induced by content or notifications of mobile phones (7).

However, current epidemiological evidence on the impact of mobile phones on sleep remains insufficient and lacks objectivity, particularly regarding the inconsistent research findings about mobile phone use and sleep time. Many studies linked mobile phone use with poorer sleep and shorter sleep time (8–13), while few studies reported minimal effects of mobile phone duration on sleep time (11, 14). Notably, two studies reported no association between the daily technology devices use and sleep (15, 16), even one study reported a positive association between mobile phone duration and sleep time (17). A critical limitation of most existing research is its reliance on self-reported mobile phone use data, which is prone to recall bias, misclassification errors, and poor correspondence with objectively measured usage (18–21). What’s more, mobile phone use is primarily characterized by mobile phone duration and unlock counts, these two key behavioral indicators capture distinct complementary facets. Mobile phone duration reflects cumulative engagement, while unlock counts reflect compulsive checking and user-device interaction patterns. Still, previous research has primarily concentrated on the relationship between mobile phone duration and sleep, the potential impact of mobile phone unlock counts on sleep has been neglected in prior studies primarily due to the difficulty of estimating the data (22). Additionally, the relationship between mobile phone use and sleep may be complex and unlikely to be a simple linear association. The digital divide and the time displacement hypothesis collectively imply a potential non-linear association between mobile phone use and sleep. The digital divide, operationalized as access disparities between mobile phone users and non-users, may prevent people from crucial health resources, resulting in mental health and sleep disparities (23). The time displacement hypothesis, by contrast, suggests that excessive mobile phone use may reduce sleep (5). These imply a potential non-linear association between mobile phone use and sleep, whereby mobile phone use may exhibit distinct effects across different exposure thresholds. However, the scarcity of objective mobile phone use data and conventional linear models often miss potential non-linear relationships between continuous mobile phone use and sleep risk.

In the present study, screenshots of mobile phone use records were used to quantify mobile phone duration and unlock counts, with the objective of evaluating their comprehensive, in-depth, and robust relationships with sleep quality and sleep time among college students.

## 2 Methods

### 2.1 Study population

In this cross-sectional investigation, we enrolled a representative sample of college students from Shaanxi Province in northwestern China. Details of the study design and investigative procedures have been reported previously (24). Briefly, 6 universities were randomly sampled from the 57 institutions in Shaanxi. Subsequently, within each participating university, 2 to 4 classes were randomly chosen from every grade across each department. All students in the selected classes were invited to participate in the survey. Overall, 20,165 undergraduate students from 559 classes across 6 universities were invited to participate. To improve data quality, the questionnaire included quality-control items consisting of simple arithmetic calculations and general knowledge questions. Participants were excluded if their questionnaires were incomplete, finished in under 500 s, or contained incorrect answers to the quality-control questions. We also excluded those who failed to submit screenshots of their mobile phone use records, or whose screenshots were too blurry or cropped to extract the required mobile phone use duration and unlock counts. After these exclusions, 16,668 students were included in the final analysis. The institutional review board associated with Xi’an Jiaotong University’s Second Affiliated Hospital granted ethical clearance for this investigation (ID: 2022-248). Digital consent documentation was completed by every student prior to formal registration.

### 2.2 Measures

#### 2.2.1 Mobile phone duration and unlock counts

In this study, objective mobile phone use was characterized by mobile phone duration and unlock counts. These measures were obtained from screenshots of each participants’ mobile phone use records. We supplied brand-specific and device-specific guidance on how to capture the screenshots and asked participants to submit images showing mobile phone duration and mobile phone unlock counts during the past week. To ensure effective data collection and standardize the data collection process, 2 weeks before the formal survey, class representatives were asked to notify students to enable the screen management function in advance and set the automatic screen lock time uniformly to 1 min. Based on the current data distribution and prior studies of mobile phone use duration (25–27), we classified participants into four groups (0 ~ 21, 21 ~ 42, 42 ~ 63, and ≥63 h/week). In accordance with the distribution of our datasets and to ensure better practical guidance, we partitioned mobile phone unlocks into quartiles, selecting integer thresholds around quartile points. Consequently, we classified participants into four groups for mobile phone unlock counts (0 ~ 50, 50 ~ 150, 150 ~ 400, and ≥400 times/week).

#### 2.2.2 Sleep quality

Sleep quality over the previous month was assessed using the Pittsburgh Sleep Quality Index (PSQI) (28). The PSQI consists of 19 items covering 7 components. Each component is scored from 0 to 3, with higher scores indicating poorer sleep. Component scores are summed to yield a global PSQI score ranging from 0 to 21, with higher total scores reflecting poorer sleep quality. A score of 8 or more indicates the presence of poor sleep among the Chinese population (29). The PSQI has shown good reliability and validity in previous population-based studies (30, 31). In the present study, the Cronbach’s *α* for the PSQI was 0.85. The PSQI was administered digitally through an online questionnaire platform and was completed only once. It was collected during the same survey session in which participants uploaded screenshots of their mobile phone use records.

#### 2.2.3 Sleep time

Sleep time was evaluated via the question: “Over the past month, how many hours did you typically spend asleep at night? This time should exclude time spent in bed without falling asleep.” Participants also reported their average monthly timings for intended bedtime, achieved sleep onset, and final awakening separately to validate the self-reported sleep time (32).

#### 2.2.4 Covariates

We used a structured questionnaire to collect data on potential confounders. Sociodemographic variables included sex, academic year, ethnicity, household registration, sibling status, and parental education level. Consistent with a previous report study (33), health-related lifestyle factors included current smoking, current drinking, physical activity, and rational diet. Students who had smoked at least one cigarette or consumed at least one glass of wine within the previous 30 days were defined as current smokers or drinkers. Physical activity was evaluated using the International Physical Activity Questionnaire Short Form was utilized to assess physical activity (34). Based on standard metabolic equivalent calculations, activity was classified as low, moderate, or high. An unhealthy diet was defined as daily consumption of red meat or intake of vegetables and fruits less than once per day.

Given the well-documented associations of depressive symptoms and social support with sleep, depressive symptoms were assessed using the Self-Rating Depression Scale (SDS) (35), whereas social support was measured with the Adolescent Social Support Scale (ASSS) (36). The SDS includes 20 items scored from 1 to 4 and evaluates emotional status during the previous week. Raw total scores were multiplied by 1.25 to obtain standardized scores, with higher values indicating more severe depressive symptoms. A standardized score >50 was used to identify depressive symptoms (37). The ASSS consists of 16 items, each rated on a 5-point scale, and higher total scores indicate stronger perceived social support. In the present study, the Cronbach’s *α* coefficients for the SDS and ASSS were 0.88 and 0.98.

### 2.3 Statistical analyses

Baseline characteristics of the participants were presented as frequency distributions for categorical variables and as means with standard deviations for continuous variables. We initially applied a Double Machine Learning (DML) framework based on the causal forest algorithm, following Chernozhukov et al. (38), to estimate the association between mobile phone use and sleep outcomes. This approach utilizes Neyman orthogonality and cross-fitting to mitigate regularization bias and over-fitting, ensuring robust estimation in the presence of complex, potentially non-linear confounding structures. We calculated the average treatment effect (ATE) to quantify the relationships of a 7-h/week increase in mobile phone duration and a 50-times/week increase in mobile phone unlock counts with PSQI scores and sleep time. We further estimated the conditional average treatment effects (CATE) using its mean and empirical 2.5th and 97.5th percentiles to capture individual-level variation (39). To examine treatment effect heterogeneity, subgroup analysis was performed by sex. The DML analysis was implemented in Python 3.9 using the CausalForestDML estimator in the EconML package (version 0.16.0), with random-forest nuisance models from scikit-learn (version 1.6.1). For the nuisance outcome and treatment models, random forest regressors were specified with 400 trees, a minimum leaf size of 5, and a fixed random seed. The causal forest was specified with 1,200 trees, a minimum leaf size of 30, unrestricted tree depth, a default subforest size of 4, continuous treatments, and random seed 202,403. A five-fold cross-fitting procedure with shuffled folds was used to obtain orthogonalized nuisance residuals and reduce overfitting. Second, to provide interpretable metrics, we complemented the DML analysis with traditional regression models. A multiple linear regression model was constructed to estimate *β* coefficients and corresponding 95% CIs for PSQI score and sleep time. Third, to characterize potential non-linear dose–response relationships, we utilized Restricted Cubic Spline (RCS) regression with three knots positioned at the 10th, 50th, and 90th percentiles. All models were adjusted for a comprehensive set of covariates, including sociodemographic information, health-related lifestyles, and psychological health. For all analyses, a 2-sided *p* < 0.05 was considered statistically significant. Regression analyses were conducted using R 4.0.2 software.

## 3 Results

### 3.1 Participant characteristics

The analysis included 16,668 college students, comprising 5,881 males (35.28%) and 10,787 females (64.72%). The participants were predominantly Han ethnicity [16,169 (97.01%)], with 4,940 (29.64%) came from one-child families, and 8,993 (53.95%) had rural household registration. The mean (SD) mobile phone duration was 48.83 (28.82) hours per week, while the mean (SD) phone unlock counts was 271.61 (291.02) times per week. In the overall sample, the mean (SD) PSQI score was 4.67 (2.98), and the mean (SD) sleep time was 441.29 (81.02) minutes per night. Baseline characteristics stratified by sleep quality are presented in Table 1.

**Table 1:** Characteristics of participants.

Characteristics	Participants	Poor sleep		*p*
		No	Yes	
Sex, *n* (%)				<0.001
Male	5,881 (35.28)	5,031 (85.55)	850 (14.45)	
Female	10,787 (64.72)	8,953 (83.00)	1834 (17.00)	
Academic year, *n* (%)				<0.001
1st	4,892 (29.35)	4,277 (87.43)	615 (12.57)	
2nd	3,907 (23.44)	3,234 (82.77)	673 (17.23)	
3rd	3,913 (23.48)	3,196 (81.68)	717 (18.32)	
4th+	3,956 (23.73)	3,277 (82.84)	679 (17.16)	
Ethnicity, *n* (%)				
Han	16,169 (97.01)	13,585 (84.02)	2,584 (15.98)	0.02
Others	499 (2.99)	399 (79.96)	100 (20.04)	
Household registration, *n* (%)				0.93
Rural	8,993 (53.95)	7,547 (83.92)	1,446 (16.08)	
Urban	7,675 (46.05)	6,437 (83.87)	1,238 (16.13)	
Sibling status, *n* (%)				0.01
No	4,940 (29.64)	4,199 (85.00)	741 (15.00)	
Yes	11,728 (70.36)	9,785 (83.43)	1943 (16.57)	
Maternal educational level, *n* (%)				0.07
Middle school or under	10,726 (64.35)	8,949 (83.43)	1777 (16.57)	
High school	3,401 (20.41)	2,872 (84.45)	529 (15.55)	
College or above	2,541 (15.24)	2,163 (85.12)	378 (14.88)	
Parental educational level, *n* (%)				<0.001
Middle school or under	9,123 (54.73)	7,564 (82.91)	1,559 (17.09)	
High school	3,697 (22.18)	3,159 (85.45)	538 (14.55)	
College or above	3,848 (23.09)	3,261 (84.75)	587 (15.25)	
Current smoking, *n* (%)				<0.001
No	14,700 (88.19)	12,505 (85.17)	2,195 (14.93)	
Yes	1968 (11.81)	1,479 (75.25)	489 (24.85)	
Current drinking, *n* (%)				<0.001
No	13,485 (80.90)	11,584 (85.90)	1901 (14.10)	
Yes	3,183 (19.10)	2,400 (75.40)	783 (24.60)	
Rational diet, *n* (%)				<0.001
Yes	1,294 (7.76)	1,178 (91.04)	116 (8.96)	
No	15,374 (92.24)	12,806 (83.30)	2,568 (16.70)	
Physical exercise, *n* (%)				<0.001
Moderate or high	4,446 (26.67)	3,860 (86.82)	586 (13.18)	
Low	12,222 (73.33)	10,124 (82.83)	2098 (17.17)	
Depressive symptoms score, mean (SD)	31.71 (8.76)	30.26 (8.01)	39.31 (8.62)	<0.001
Social support score, mean (SD)	67.29 (14.98)	68.41 (14.73)	61.45 (14.94)	<0.001
Mobile phone duration (hour/week), *n* (%)				<0.001
0 ~ 21	3,707 (22.24)	3,168 (85.56)	539 (14.54)	
21 ~ 42	2,818 (16.91)	2,447 (86.83)	371 (13.17)	
42 ~ 63	4,322 (25.93)	3,687 (85.31)	635 (14.69)	
≥63	5,821 (34.92)	4,682 (80.43)	1,139 (19.57)	
Mobile phone duration (hour/week), mean (SD)	48.83 (28.82)	48.05 (28.59)	52.88 (29.65)	0.02
Mobile phone unlock counts (times/week), n (%)				<0.001
0 ~ 50	3,525 (21.15)	3,092 (87.72)	433 (12.28)	
50 ~ 150	4,766 (28.59)	4,046 (84.89)	720 (15.11)	
150 ~ 400	4,091 (24.54)	3,382 (82.67)	709 (17.33)	
≥400	4,286 (25.71)	3,464 (80.82)	822(19.18)	
Mobile phone unlock counts (times/week), mean (SD)	271.61 (291.02)	264.00 (286.97)	311.23 (308.32)	<0.001

SD, standard deviation.

### 3.2 Associations between mobile phone use and sleep: DML analysis

The DML model was employed to estimate the ATE of mobile phone use on sleep metrics while accounting for sociodemographic information, health-related lifestyles, and psychological health (Table 2). Each 7-h increase in weekly mobile phone duration was significantly associated with an increase in the PSQI score (*ATE* = 0.03, *95%CI*: 0.02 to 0.03) and less sleep time (*ATE* = −4.76, *95%CI*: −6.13 to −3.65). Similarly, a 50-unit increase in weekly mobile phone unlock counts was associated with a higher PSQI score (*ATE* = 0.42, *95%CI*: 0.21, 0.63) and less sleep time (*ATE* = −0.89, *95%CI*: −1.32 to −0.46).

**Table 2:** Estimated average treatment effects of mobile phone duration and unlock counts on the sleep quality and sleep time.

Mobile phone use	PSQI score		Sleep time	
	ATE	CATE	ATE	CATE
Mobile phone duration, 7 h/week	0.03 (0.02, 0.03)	0.03(0.01, 0.05)	−4.76 (−6.13, −3.65)	−4.76 (−8.84, −0.68)
Male	0.03 (0.01, 0.05)		−4.71 (−7.70, −3.58)	
Female	0.03 (0.01, 0.05)		−4.79 (−6.64, −3.15)	
Mobile phone unlock counts, 50 times/week	0.42 (0.21, 0.63)	0.42 (0.21, 0.63)	−0.89 (−1.32, −0.46)	−0.88 (−1.30, −0.45)
Male	0.36 (0.17, 0.55)		−0.85 (−1.26, −0.45)	
Female	0.48 (0.25, 0.71)		−0.92 (−1.40, −0.44)	

Adjusted for sex, academic year, ethnicity, household registration, sibling status, parental education level, smoking, drinking, physical activity, diet, depressive symptoms, and social support.

PSQI, Pittsburgh Sleep Quality Index; ATE, average treatment effect; CATE, conditional average treatment effect.

Subgroup analysis revealed the sex-specific heterogeneity in the associations between mobile phone use and sleep. The detrimental associations between mobile phone unlock counts and sleep were more pronounced in females than in males. Specifically, females exhibited a higher ATE for PSQI scores [(*ATE* = 0.48, *95%CI*: 0.25 to 0.71) vs. (*ATE* = 0.36, *95% CI*: 0.17 to 0.55)] and a marginally greater reduction in sleep time [(*ATE* = −0.92, *95%CI*: −1.40 to −0.44) vs. (*ATE* = −0.85, *95%CI*: −1.26 to −0.45)] compared to males. In contrast, these gender disparities were notably attenuated when examining mobile phone duration. These associations with PSQI scores were identical across genders (*ATE* = 0.03, *95%CI*: 0.01 to 0.05 for both). However, a marginal difference persisted for sleep time, with females (*ATE* = −4.79, *95%CI*: −6.64 to −3.15) experiencing a slightly larger decrease than males (*ATE* = −4.71, *95%CI*: −7.70 to −3.58).

### 3.3 Associations between mobile phone use and sleep: linear regression analysis

After adjustment for the same set of confounders used in the DML framework, traditional linear regression analyses confirmed significant relationships (Table 3). Relative to participants reporting 0–21 h/week of mobile phone duration, those with ≥63 h/week had significantly higher PSQI score (*β* = 0.34, *95%CI*: 0.23 to 0.44). In contrast, those with 21–42 h/week (*β* = −0.05, *95%CI*: −0.17 to 0.08) and 42–63 h/week (*β* = 0.10, *95%CI*: −0.01 to 0.22) of mobile phone duration exhibited no significant increase in PSQI score. For sleep time, compared with the 0–21 h/week group, participants with ≥63 h/week of mobile phone duration slept fewer minutes per night (*β* = −3.49, *95%CI*: −6.88 to −0.10). By contrast, mobile phone duration of 21–42 h/week (*β* = 4.72, *95%CI*: 0.74 to 8.70) and 42–63 h/week (*β* = 3.66, *95%CI*: 0.05 to 7.27) were linked to longer sleep time.

**Table 3:** Associations of mobile phone duration, and unlock counts with sleep quality and sleep time.

Mobile phone use	*β (95%CI)*	
	PSQI score	Sleep time
Mobile phone duration (hour/week)		
0 ~ 21	Reference	Reference
21 ~ 42	−0.05 (−0.17 to 0.08)	4.72 (0.74, 8.70)
42 ~ 63	0.10 (−0.01 to 0.22)	3.66 (0.05, 7.27)
≥63	0.34 (0.23 to 0.44)	−3.49 (−6.88, −0.10)
Mobile phone unlock counts (times/week)		
0 ~ 50	Reference	Reference
50 ~ 150	0.07 (−0.04 to 0.19)	4.63 (1.05, 8.21)
150 ~ 400	0.36 (0.25 to 0.48)	−3.06 (−6.75, 0.63)
≥400	0.52 (0.40 to 0.63)	−9.90 (−13.57, −6.22)

Adjusted for sex, academic year, ethnicity, household registration, sibling status, parental education level, smoking, drinking, physical activity, diet, depressive symptoms, and social support.

*β*, regression coefficient; CI, confidence interval; PSQI, Pittsburgh Sleep Quality Index.

Compared with participants with 0–50 times/week of unlock counts, those with 150–400 unlocks/week (*β* = 0.36, *95%CI*: 0.25 to 0.48) and ≥400 times/week (*β* = 0.52, *95%CI*: 0.40 to 0.63) showed progressively higher PSQI scores, although the association for 50–150 times/week (*β* = 0.07, *95%CI*: −0.04 to 0.19) was not statistically significant. For sleep time, compared with the 0–50 times/week of unlock counts group, those with 50–150 times/week of unlock counts had a significantly longer sleep time (*β* = 4.63, *95%CI*: 1.05 to 8.21), those with ≥400 times/week of unlock counts had a significantly shorter sleep time (*β* = −9.90, *95%CI*: −13.57 to −6.22). No significant association with sleep time was observed among participants with 150 ~ 400 times/week of unlock counts (*β* = −3.06, *95%CI*: −6.75 to 0.63).

### 3.4 Non-linear associations between mobile phone use and sleep: RCS analysis

**Figure 1:**
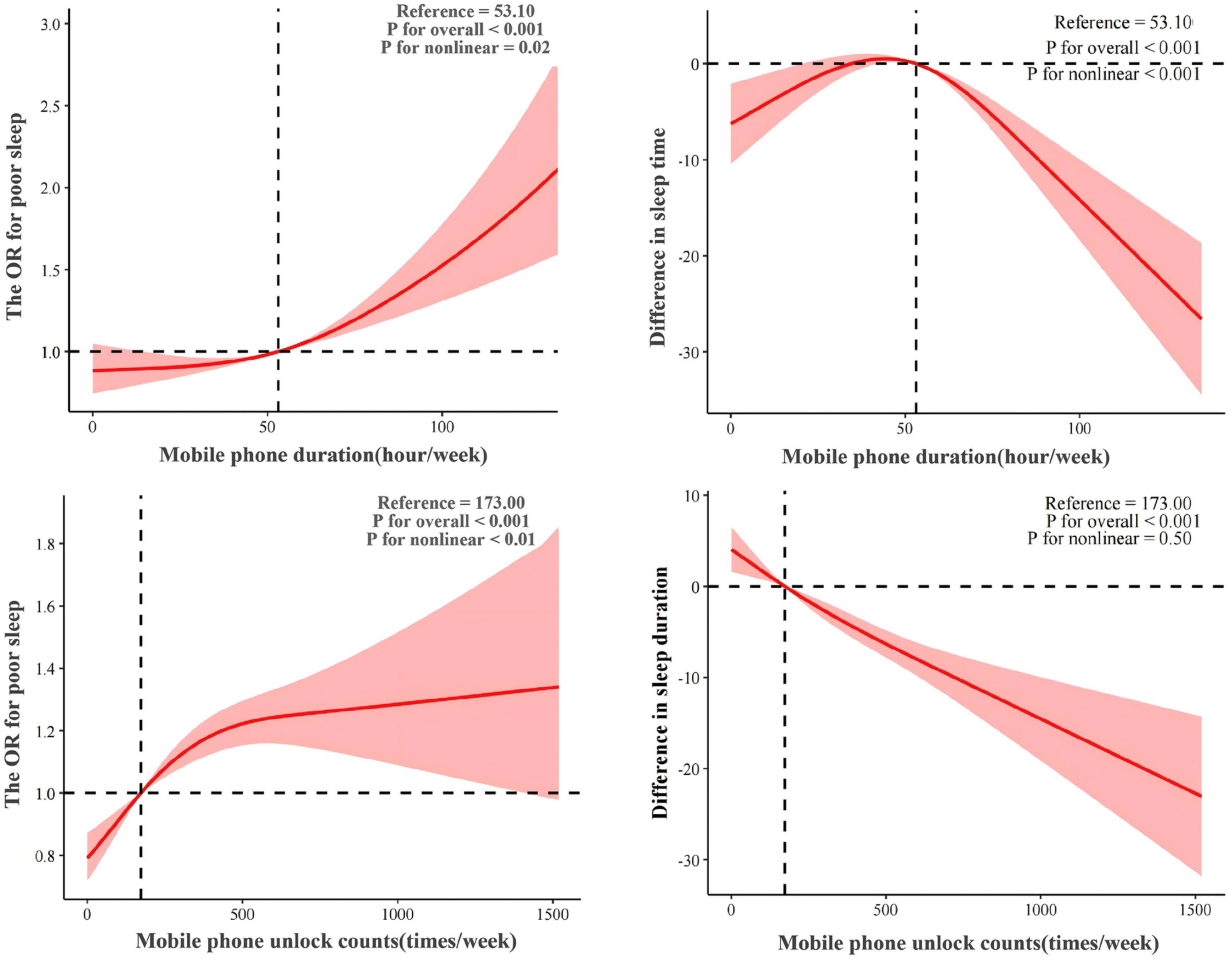
Restricted cubic splines regression analyses of mobile phone duration, and unlock counts with odds of poor sleep and reduction of sleep time. Note: adjusted for sex, academic year, ethnicity, household registration, sibling status, parental education level, smoking, drinking, physical activity, diet, depressive symptoms, and social support.

RCS models further elucidated the complex non-linear nature of these associations (Figure 1). A significant non-linear association was observed between mobile phone duration and the risk of poor sleep (*P* for non-linearity = 0.02). Furthermore, mobile phone duration showed a significant inverted U-shaped nonlinear relationship with sleep time (*P* for non-linearity<0.001). Regarding mobile phone unlock counts, a significant non-linear association was observed between mobile phone unlock counts and odds of poor sleep (*P* for non-linearity<0.01), while its relationship with sleep time followed a predominantly monotonic decline (*P* for non-linearity = 0.50).

## 4 Discussion

In this large-scale study, we provide robust evidence linking both mobile phone duration and unlock counts to sleep quality and duration. DML revealed that increased mobile phone duration and higher unlock frequencies are independently associated with shortened sleep time and elevated PSQI scores. Furthermore, RCS analyses indicated non-linear relationships of mobile phone duration with both sleep time and odds of poor sleep, whereas an inverted U-shaped association was observed between mobile phone duration and sleep time. Mobile phone unlock counts exhibited a non-linear relationship with sleep time, but a predominantly monotonic inverse relationship with the risk of poor sleep.

### 4.1 Comparison with other studies

Despite the expanding body of literature regarding mobile phone use and sleep, previous research has predominantly centered on subjective constructs, such as mobile phone addiction. Empirical evidence linking objective duration and unlock counts to sleep quality and time remains remarkably scarce. In particular, the distinct impact of mobile phone unlock counts on sleep outcomes represents a critical, yet largely unexplored, gap in current research. Most published studies, however, rely on participants’ self-reported data on mobile phone use. For example, a study involving 804 college students found a negative association between self-reported mobile phone use and sleep quality (9). Another cross-sectional study with 1,101 Spanish adolescents reported that the time spent on mobile phones was negatively associated with sleep time (*β* = −0.306; *p* < 0.001) (40). Consistently, a systematic review incorporating 17 studies with a total of 36,485 participants further validated that excessive smartphone use is closely linked to deteriorated self-perceived sleep quality, sleep insufficiency, and prolonged sleep latency (41). In contrast, a Danish adult study found that self-reported daily mobile phone use was associated with an average 0.15-point higher in sleep quality score (42), while mobile phone unlock counts showed no association with either sleep quality or sleep time. The SPUTNIC study, including 121 participants with daily data, found no associations of sleep quality and duration with either cordless, mobile phone calls, or mobile phone duration (16), though mobile phone unlock data were not collected. Nevertheless, the above research relying on participants’ self-reported mobile phone use is vulnerable to recall bias, misclassification, and may not be appropriate for people with psychological problems (18–21).

To our knowledge, only a small body of literature has utilized objective measures to investigate how average mobile phone duration is associated with youth sleep (14, 43–45), and only one study assesses how average objective mobile phone unlock counts are associated with youth sleep (14). One study continuously monitored mobile phone duration for 30 days using a mobile phone application in 136 participants, after adjusting for age, sex, ethnicity, and sleep apnea history, it found that longer average mobile phone duration was associated with shorter sleep time and reduced sleep efficiency (43). Another investigation enrolling 269 participants further demonstrated that elevated smartphone engagement was linked to shortened sleep duration, amplified sleep disruption, and delayed bedtime (45). A Chinese Hong Kong-based study installed an app to track participants’ mobile phone usage, and found that each additional minute of mobile phone duration was associated with a 0.07-min reduction in total sleep time that night, but was not associated with sleep efficiency or awakenings after sleep onset (44). Notably, all the aforementioned studies focused exclusively on mobile phone screen impact, and consensus remains lacking regarding their impact on sleep quality. Only one recent study (14), which included 7,499 users, found that mobile phone duration and unlock counts emerged as statistically significant predictors of sleep time, although the magnitude of the association was small.

Our findings align closely with this research, highlighting that objectively measured mobile phone duration and unlock counts exert a relatively significant impact on sleep quality, and also have a certain effect on sleep time. The current study provides an important contribution to the extant literature, as there is an extreme deficiency in objectively measured mobile phone duration and unlock counts data, as well as combined mobile phone duration and unlock counts on sleep, especially among Chinese, whose mobile phone ownership and addiction rates are at the forefront globally (46).

### 4.2 Non-linear association between mobile phone use and sleep

Moreover, evidence regarding the non-linear association between mobile phone use and sleep remains limited. We found non-linear associations of mobile phone duration and unlock counts with sleep time, as well as a non-linear relationship between mobile phone duration and odds of poor sleep. Notably, an inverted U-shaped relationship was observed between mobile phone duration and sleep time. These findings underscore the multifaceted and complex nature of the association between mobile phone use and sleep. On one hand, the digital divide between mobile phone users and non-users may prevent people from accessing crucial health resources, resulting in mental health and sleep disparities (23). Consistent with the conservation of resources theory (47), technological advances such as mobile phones play an integral role in acquiring and sustaining diverse psychosocial resources. Consequently, low-to-moderate mobile phone use may serve as a proxy for individuals’ ability to navigate stressors, maintain resilience, and preserve sleep quality (48–50). Aligning with these perspectives, RCS analyses in the present study suggested that moderate mobile phone duration does not necessarily demonstrate an adverse association with sleep. A plausible explanation is that moderate use is linked to the acquisition of social information and the maintenance of psychosocial resources, potentially correlating with positive factors such as social support and convenient information accessibility in certain contexts. Conversely, very low levels of mobile phone use might be associated with restricted social connectivity and reduced information access, which could indirectly relate to suboptimal sleep. On the other hand, high-intensity mobile phone use may be negatively associated with sleep through multiple biological and behavioral pathways. First, prolonged mobile phone use involves increased exposure to screen-derived light (6). which has been extensively linked to delayed melatonin secretion, disrupted circadian rhythms, and subsequently poorer sleep quality and curtailed sleep duration. Second, the time displacement hypothesis posits that excessive mobile phone use displaces time otherwise spent sleeping (5). Brosnan et al. (11) suggested that this association is primarily driven by delayed sleep onset rather than direct photic mechanisms of blue light or interactive content, as evidenced by minimal associations with sleep latency or nocturnal wakefulness. Finally, the psychological and physiological arousal elicited by engaging, entertaining, or distressing content across various applications may also be associated with an impaired ability to initiate and maintain sleep (7, 51). Nevertheless, interpretations of the observed inverted U-shaped relationship must be made with caution. Although this study utilized objectively measured mobile phone data, we did not collect information on specific use content, timing of use, bedtime engagement, notification exposure, ambient light exposure, psychological stress, loneliness, social support, mobile phone dependence, or underlying motivations for use. Therefore, the proposed explanations should be viewed as plausible hypotheses derived from existing literature and theoretical frameworks, rather than causal mechanisms directly validated by this cross-sectional data.

In contrast to the non-linear relationship observed between mobile phone total duration and odds of poor sleep, we found that PSQI score decreased monotonically as mobile phone unlock counts increased. We posit that these divergent dose–response trajectories stem from the fundamentally distinct behavioral phenotypes and underlying psychological drivers captured by these two objective metrics. Specifically, mobile phone total duration reflects cumulative engagement, including active interaction and passive media exposure, and mainly functions as an indicator of overall usage intensity. Conversely, unlock counts more directly measure compulsive phone-checking behavior, which is often motivated by notification reliance or nomophobia, and represents a central feature of mobile phone addiction (52). Neurobiologically, frequent unlocking is intrinsically tied to reward-seeking circuitry, particularly dopamine-driven. This direct link to addictive pathways likely triggers sustained central nervous system arousals, thereby disrupting the physiological readiness required for sleep onset and maintenance (53). A systematic review encompassing 14 studies found that mobile phone use dependence was associated with a significantly higher risk of poor sleep (*OR* = 2.19, *95% CI*: 1.79 to 2.67) (54). More recently, a study of college students reported positive associations of mobile phone addiction with bedtime procrastination and sleep disorders (55). Although these earlier studies predominantly relied on subjective scales and did not characterize non-linear dynamic trajectories, they consistently highlight the detrimental impact of addictive or dependent mobile phone use on sleep. As mobile phone unlock counts serve as a highly objective proxy for this addictive behavior, its direct and continuous erosion of sleep quality logically explains the strictly monotonic decrease observed in our data.

### 4.3 Sex differences of the association between mobile phone use and sleep

In this study, the ATE for the association of mobile phone unlock counts with sleep quality and duration was more pronounced in females than in males, whereas no prominent sex differences were observed regarding the associations between mobile phone duration and sleep. Previous research has demonstrated that males tend to favor gaming applications, whereas females commonly gravitate toward social media platforms (40). Frequent social media engagement corresponds to a higher frequency of mobile phone unlocking, which may account for the observed sex disparities in the association between unlock counts and sleep parameters. As a central subcategory of mobile phone addiction and a primary driver of female mobile phone dependency, social media addiction has been confirmed to be associated with sleep (56–58). Consistent with the current study, a recent study has reported that social networking application use was significantly associated with sleep-related problems exclusively among females, with no significant association observed in males (40). This implies that sex disparities in the association between mobile phone use and sleep may stem from females’ preference for social media. This study did not assess sex-related differences in the application preferences of participants. Therefore, future studies should collect more detailed information on applications used to better examine sex-specific patterns and their potential underlying mechanisms.

### 4.4 Limitations

First, given the cross-sectional design, the direction of the association remains uncertain. It cannot be determined whether excessive mobile phone use contributes to poorer sleep quality and shorter sleep duration, nor can we exclude the possibility that poor sleep leads to increased mobile phone use. Furthermore, the absence of information on physician-diagnosed sleep disorders could introduce residual confounding or reverse causation. This highlights the need for future longitudinal investigations that account for the clinical history of sleep disorders. Second, although the models adjusted for a broad range of factors, residual confounding may still have persisted and influenced the results. Third, sleep was assessed using a retrospective questionnaire during the past month, which may introduce the possibility of recall bias. Fourth, although mobile phone duration and unlock counts were measured objectively, this study did not differentiate functional categories of mobile phone use, which may have differential associations with sleep. Moreover, we did not evaluate nighttime mobile phone use or nighttime unlock events. Prior evidence has demonstrated that excessive bedtime mobile phone engagement exhibits more pronounced associations with sleep (59, 60). The absence of bedtime mobile phone use data in this study may have limited the ability to fully characterize sleep disturbances related to nighttime mobile phone use. Future studies should use passive sensing applications or device-level logs to capture nighttime mobile phone use, classify mobile phone use by functional category, and further examine the associations of these usage patterns with sleep. Fifth, the differing recall periods for mobile phone use (preceding week) and sleep (preceding month) represent another limitation. Although the past-week assessment window for mobile phone use was within the PSQI assessment period, and empirical evidence (61) suggests that one-week objective mobile phone use data can reliably reflect habitual patterns of mobile phone use, the differing time frames may have potentially biased the observed associations.

## 5 Conclusion

This study of 16,668 college students using DML and traditional linear regression showed that both mobile phone duration and unlock counts were independently associated with poor sleep quality and short sleep time. RCS analyses further indicated non-linear relationships of mobile phone duration with sleep time and odds of poor sleep, whereas an inverted U-shaped association was observed between mobile phone duration and sleep time. Mobile phone unlock counts exhibited a non-linear relationship with sleep time, but a predominantly monotonic inverse relationship with the risk of poor sleep.

## Data Availability

The raw data supporting the conclusions of this article will be made available by the authors, without undue reservation.
